# *N*-(2,3-Dimethyl­phen­yl)-2-methyl­benzamide

**DOI:** 10.1107/S1600536811050732

**Published:** 2011-11-30

**Authors:** Vinola Z. Rodrigues, B. Thimme Gowda, Július Sivý, Viktor Vrábel, Jozef Kožíšek

**Affiliations:** a Department of Chemistry, Mangalore University, Mangalagangotri 574 199, Mangalore, India; b Institute of Mathematics and Physics, Faculty of Mechanical Engineering, Slovak Technical University, Namestie slobody 17, SK-812 31 Bratislava, Slovak Republic; c Institute of Physical Chemistry and Chemical Physics, Faculty of Chemical and Food Technology, Slovak Technical University, Radlinského 9, SK-812 37 Bratislava, Slovak Republic

## Abstract

In the title compound, C_16_H_17_NO, the two aromatic rings make a dihedral angle of 5.9 (2)°, while the central amide core –NH—C(=O)– is twisted by 44.0 (3) and 47.1 (3)° out of the planes of the 2,3-dimethyl­phenyl and 2-methyl­phenyl rings, respectively. In the crystal, N—H⋯O hydrogen bonds link the mol­ecules into infinite chains running along the *b* axis.

## Related literature

For the preparation of the title compound, see: Gowda *et al.* (2003 ▶). For our studies on the effects of substituents on the structures and other aspects of *N*-(ar­yl)-amides, see: Bowes *et al.* (2003 ▶); Gowda *et al.* (2000 ▶); Saeed *et al.* (2010 ▶) on *N*-(ar­yl)-methane­sulfonamides, see: Jayalakshmi & Gowda (2004 ▶), on *N*-(ar­yl)-aryl­sulfonamides, see: Shetty & Gowda (2005 ▶) and on *N*-chloro­aryl­amides, see: Gowda *et al.* (1996 ▶).
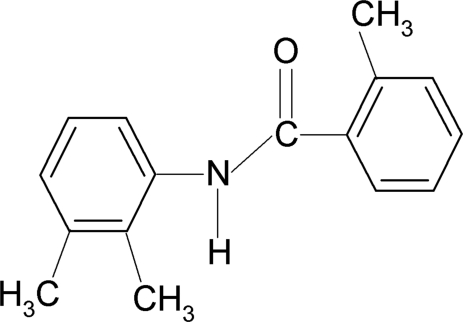


## Experimental

### 

#### Crystal data

C_16_H_17_NO
*M* *_r_* = 239.31
Monoclinic, 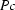

*a* = 5.8092 (4) Å
*b* = 4.9253 (2) Å
*c* = 23.1887 (12) Å
β = 94.229 (5)°
*V* = 661.67 (6) Å^3^
*Z* = 2
Mo *K*α radiation
μ = 0.08 mm^−1^
*T* = 295 K
0.50 × 0.30 × 0.10 mm

#### Data collection

Oxford Diffractio Xcalibur System diffractometer
Absorption correction: multi-scan (*CrysAlis RED*; Oxford Diffraction, 2009 ▶) *T*_min_ = 0.962, *T*_max_ = 0.993
9487 measured reflections
1162 independent reflections
1021 reflections with *I* > 2σ(*I*)
*R*_int_ = 0.043

#### Refinement

*R*[*F* ^2^ > 2σ(*F* ^2^)] = 0.068
*wR*(*F* ^2^) = 0.208
*S* = 1.10
1162 reflections
170 parameters
3 restraints
H atoms treated by a mixture of independent and constrained refinement
Δρ_max_ = 0.29 e Å^−3^
Δρ_min_ = −0.23 e Å^−3^

### 

Data collection: *CrysAlis CCD* (Oxford Diffraction, 2009 ▶); cell refinement: *CrysAlis CCD*; data reduction: *CrysAlis RED* (Oxford Diffraction, 2009 ▶); program(s) used to solve structure: *SHELXS97* (Sheldrick, 2008 ▶); program(s) used to refine structure: *SHELXL97* (Sheldrick, 2008 ▶); molecular graphics: *DIAMOND* (Brandenburg, 2002 ▶) and *ORTEP-3* (Farrugia, 1997 ▶); software used to prepare material for publication: *SHELXL97*, *PLATON* (Spek, 2009 ▶) and *WinGX* (Farrugia, 1999 ▶).

## Supplementary Material

Crystal structure: contains datablock(s) I, global. DOI: 10.1107/S1600536811050732/bt5725sup1.cif

Structure factors: contains datablock(s) I. DOI: 10.1107/S1600536811050732/bt5725Isup2.hkl

Supplementary material file. DOI: 10.1107/S1600536811050732/bt5725Isup3.cml

Additional supplementary materials:  crystallographic information; 3D view; checkCIF report

## Figures and Tables

**Table 1:** Hydrogen-bond geometry (Å, °)

*D*—H⋯*A*	*D*—H	H⋯*A*	*D*⋯*A*	*D*—H⋯*A*
N1—H1⋯O1^i^	0.86 (1)	2.23 (5)	2.903 (6)	136 (6)

Symmetry code: (i) 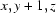.

**Fig. 1.**
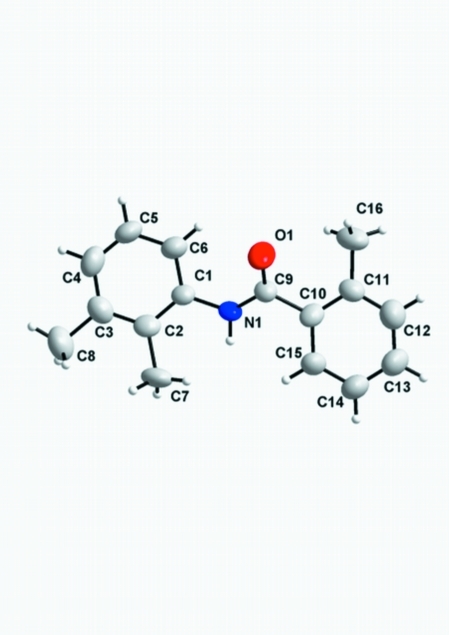
Molecular structure of the title compound with the atomic numbering scheme. Displacement ellipsoids are drawn at the 50% probability level. H atoms are represented as small spheres of arbitrary radii.

**Fig. 2.**
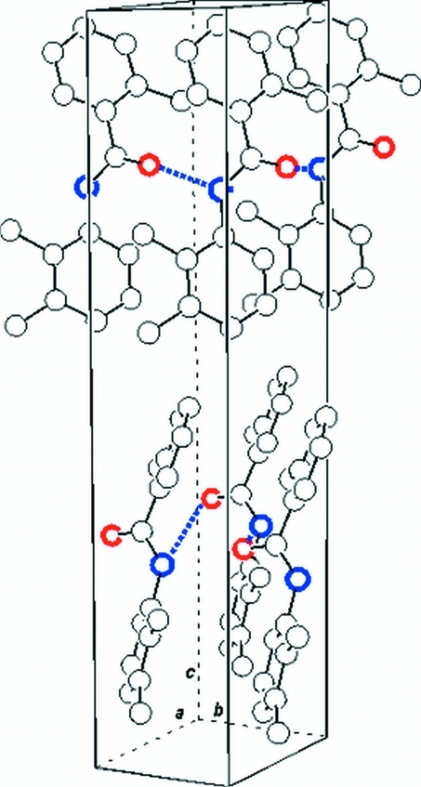
Packing view of the title compound. Molecular chains along b axis are generated by N–H···O hydrogen bonds which are shown by dashed lines. H atoms not involved in H-bonding have been omitted.

## supplementary crystallographic information

### Comment

The amide and sulfonamide moieties are the constituents of many biologically significant compounds. As part of our studies on the substituent effects on the structures and other aspects of *N*-(aryl)-amides (Bowes *et al.*, 2003; Gowda *et al.*, 2000; Saeed *et al.*, 2010), *N*-(aryl)-methanesulfonamides (Jayalakshmi & Gowda, 2004), *N*-(aryl)-arylsulfonamides (Shetty & Gowda, 2005) and *N*-chloro-arylsulfonamides (Gowda *et al.*, 1996), in the present work, the crystal structure of *N*-(2,3-dimethylphenyl)-2-methylbenzamide (I) has been determined (Fig. 1).

In (I), the two aromatic rings make the dihedral angle of 5.9 (2)°, while the central amide core –NH—C(=O)– is twisted by 44.0 (3) ° and 47.1 (3)° out of the planes of the 2,3-dimethylphenyl and 2-methylphenyl rings, respectively.

Further, the *ortho*-methyl group in the benzoyl ring is positioned *syn* to the C=O bond and so also the *ortho*- and *meta*- methyl groups in the anilino ring to the N—H bond, while the N—H and C=O bonds in the C—NH—C(O)—C segment are *anti* to each other.

In the crystal structure, intermolecular N—H···O hydrogen bonds link the molecules into infinite chains running along the *b*-axis. Part of the crystal structure is shown in Fig. 2.

### Experimental

The title compound was prepared according to the method described by Gowda *et al.* (2003). The purity of the compound was checked by determining its melting point. It was characterized by recording its infrared and NMR spectra. Plate like colourless single crystals of the title compound were obtained by slow evaporation of an ethanol solution of the compound (0.5 g in about 30 ml of ethanol) at room temperature.

### Refinement

All hydrogen atoms except amide H atom were placed in calculated positions with C–H distances in the range 0.93–0.96 Å and constrained to ride on their parent atoms. The amide H atom was found in a difference map and was refined with the N—H distance restrained to 0.86 (3) Å. The *U*ĩso~(H) values were set at 1.2*U*_eq_(C-aromatic, N) or 1.5*U*_eq_(C-methyl). In the absence of significant anomalous scattering, the absolute structure could not be reliably determined and then the Friedel pairs were merged and any references to the Flack parameter were removed.

### Crystal data

**Table:**

C_16_H_17_NO	*F*(000) = 256
*M**_r_* = 239.31	*D*_x_ = 1.201 Mg m^−^^3^
Monoclinic, *P**c*	Mo *K*α radiation, λ = 0.71073 Å
Hall symbol: P -2yc	Cell parameters from 6163 reflections
*a* = 5.8092 (4) Å	θ = 3.5–25.0°
*b* = 4.9253 (2) Å	µ = 0.08 mm^−^^1^
*c* = 23.1887 (12) Å	*T* = 295 K
β = 94.229 (5)°	Plate, colourless
*V* = 661.67 (6) Å^3^	0.50 × 0.30 × 0.10 mm
*Z* = 2	

### Data collection

**Table:**

Oxford Diffractio Xcalibur System diffractometer	1162 independent reflections
Radiation source: fine-focus sealed tube	1021 reflections with *I* > 2σ(*I*)
graphite	*R*_int_ = 0.043
Detector resolution: 0 pixels mm^-1^	θ_max_ = 25.0°, θ_min_ = 3.5°
\j scans, and ω scans with κ offsets	*h* = −6→6
Absorption correction: multi-scan (*CrysAlis RED*; Oxford Diffraction, 2009)	*k* = −5→5
*T*_min_ = 0.962, *T*_max_ = 0.993	*l* = −27→27
9487 measured reflections	

### Refinement

**Table:**

Refinement on *F*^2^	Primary atom site location: structure-invariant direct methods
Least-squares matrix: full	Secondary atom site location: difference Fourier map
*R*[*F*^2^ > 2σ(*F*^2^)] = 0.068	Hydrogen site location: inferred from neighbouring sites
*wR*(*F*^2^) = 0.208	H atoms treated by a mixture of independent and constrained refinement
*S* = 1.10	*w* = 1/[σ^2^(*F*_o_^2^) + (0.122*P*)^2^ + 0.430*P*] where *P* = (*F*_o_^2^ + 2*F*_c_^2^)/3
1162 reflections	(Δ/σ)_max_ < 0.001
170 parameters	Δρ_max_ = 0.29 e Å^−^^3^
3 restraints	Δρ_min_ = −0.23 e Å^−^^3^

### Special details

**Table:**

Geometry. All e.s.d.'s (except the e.s.d. in the dihedral angle between two l.s. planes) are estimated using the full covariance matrix. The cell e.s.d.'s are taken into account individually in the estimation of e.s.d.'s in distances, angles and torsion angles; correlations between e.s.d.'s in cell parameters are only used when they are defined by crystal symmetry. An approximate (isotropic) treatment of cell e.s.d.'s is used for estimating e.s.d.'s involving l.s. planes.
Refinement. Refinement of *F*^2^ against ALL reflections. The weighted *R*-factor *wR* and goodness of fit *S* are based on *F*^2^, conventional *R*-factors *R* are based on *F*, with *F* set to zero for negative *F*^2^. The threshold expression of *F*^2^ > σ(*F*^2^) is used only for calculating *R*-factors(gt) *etc*. and is not relevant to the choice of reflections for refinement. *R*-factors based on *F*^2^ are statistically about twice as large as those based on *F*, and *R*- factors based on ALL data will be even larger.

### Fractional atomic coordinates and isotropic or equivalent isotropic displacement parameters (Å^2^)

**Table:**

	*x*	*y*	*z*	*U*_iso_*/*U*_eq_	
C1	0.7345 (9)	0.2228 (10)	0.2068 (2)	0.0440 (13)	
C2	0.9138 (11)	0.3524 (12)	0.1826 (3)	0.0507 (14)	
C3	0.9528 (11)	0.3043 (14)	0.1241 (3)	0.0582 (16)	
C4	0.8099 (14)	0.1135 (15)	0.0929 (3)	0.069 (2)	
H4	0.8364	0.0739	0.0548	0.083*	
C5	0.6384 (13)	−0.0099 (15)	0.1175 (3)	0.0661 (19)	
H5	0.5456	−0.1324	0.0959	0.079*	
C6	0.5950 (12)	0.0403 (13)	0.1744 (3)	0.0582 (16)	
H6	0.4740	−0.0471	0.1909	0.070*	
C7	1.0716 (12)	0.5454 (14)	0.2186 (4)	0.069 (2)	
H7A	1.2242	0.5380	0.2054	0.104*	
H7B	1.0768	0.4924	0.2585	0.104*	
H7C	1.0130	0.7272	0.2145	0.104*	
C8	1.1354 (16)	0.4573 (18)	0.0958 (4)	0.081 (2)	
H8A	1.1608	0.3746	0.0593	0.122*	
H8B	1.2761	0.4538	0.1203	0.122*	
H8C	1.0868	0.6420	0.0896	0.122*	
C9	0.6479 (10)	0.0862 (11)	0.3048 (2)	0.0446 (13)	
C10	0.6090 (11)	0.1940 (11)	0.3638 (2)	0.0485 (14)	
C11	0.4282 (10)	0.1026 (12)	0.3934 (3)	0.0520 (15)	
C12	0.4113 (16)	0.2012 (17)	0.4479 (3)	0.075 (2)	
H12	0.2908	0.1411	0.4689	0.090*	
C13	0.5661 (15)	0.3872 (16)	0.4731 (3)	0.072 (2)	
H13	0.5472	0.4519	0.5102	0.086*	
C14	0.7478 (14)	0.4764 (15)	0.4435 (3)	0.0680 (19)	
H14	0.8540	0.5996	0.4603	0.082*	
C15	0.7695 (10)	0.3809 (13)	0.3890 (3)	0.0528 (15)	
H15	0.8913	0.4397	0.3683	0.063*	
C16	0.2533 (14)	−0.0985 (15)	0.3695 (4)	0.074 (2)	
H16A	0.1224	−0.0972	0.3925	0.111*	
H16B	0.3206	−0.2766	0.3703	0.111*	
H16C	0.2046	−0.0508	0.3304	0.111*	
N1	0.6911 (9)	0.2757 (9)	0.2654 (2)	0.0478 (12)	
H1	0.756 (11)	0.427 (8)	0.276 (3)	0.06 (2)*	
O1	0.6366 (11)	−0.1558 (8)	0.2943 (2)	0.0708 (14)	

### Atomic displacement parameters (Å^2^)

**Table:**

	*U*^11^	*U*^22^	*U*^33^	*U*^12^	*U*^13^	*U*^23^
C1	0.054 (3)	0.029 (2)	0.049 (3)	0.002 (2)	0.003 (2)	−0.003 (2)
C2	0.059 (4)	0.029 (3)	0.063 (4)	0.005 (2)	0.000 (3)	0.001 (2)
C3	0.057 (4)	0.052 (4)	0.066 (4)	0.005 (3)	0.008 (3)	0.015 (3)
C4	0.098 (6)	0.065 (4)	0.044 (3)	0.012 (4)	0.007 (4)	0.003 (3)
C5	0.090 (5)	0.056 (4)	0.050 (4)	−0.014 (4)	−0.005 (3)	−0.013 (3)
C6	0.072 (4)	0.047 (3)	0.055 (4)	−0.010 (3)	−0.001 (3)	−0.006 (3)
C7	0.062 (4)	0.052 (4)	0.094 (5)	−0.021 (3)	0.005 (3)	−0.012 (4)
C8	0.094 (5)	0.077 (5)	0.076 (5)	0.006 (5)	0.025 (4)	0.023 (4)
C9	0.053 (3)	0.032 (3)	0.049 (3)	0.002 (2)	0.007 (2)	0.000 (2)
C10	0.068 (4)	0.031 (3)	0.046 (3)	0.008 (3)	−0.002 (3)	0.002 (2)
C11	0.056 (4)	0.036 (3)	0.065 (4)	0.001 (3)	0.007 (3)	0.003 (3)
C12	0.091 (6)	0.070 (5)	0.064 (4)	−0.002 (4)	0.015 (4)	0.009 (4)
C13	0.103 (6)	0.061 (4)	0.049 (4)	−0.006 (4)	−0.001 (4)	−0.001 (3)
C14	0.086 (5)	0.059 (4)	0.058 (4)	−0.008 (4)	0.001 (3)	−0.010 (3)
C15	0.059 (4)	0.048 (3)	0.051 (3)	−0.006 (3)	−0.001 (3)	0.002 (3)
C16	0.072 (5)	0.048 (4)	0.104 (6)	−0.012 (3)	0.011 (4)	−0.008 (4)
N1	0.060 (3)	0.028 (2)	0.056 (3)	−0.008 (2)	0.012 (2)	−0.004 (2)
O1	0.120 (4)	0.028 (2)	0.066 (3)	0.003 (2)	0.019 (2)	0.001 (2)

### Geometric parameters (Å, °)

**Table:**

C1—C2	1.376 (9)	C9—O1	1.217 (7)
C1—C6	1.392 (8)	C9—N1	1.342 (7)
C1—N1	1.424 (7)	C9—C10	1.502 (8)
C2—C3	1.412 (9)	C10—C11	1.373 (9)
C2—C7	1.526 (9)	C10—C15	1.406 (9)
C3—C4	1.416 (10)	C11—C12	1.362 (10)
C3—C8	1.491 (11)	C11—C16	1.496 (9)
C4—C5	1.330 (11)	C12—C13	1.383 (11)
C4—H4	0.9300	C12—H12	0.9300
C5—C6	1.385 (10)	C13—C14	1.374 (11)
C5—H5	0.9300	C13—H13	0.9300
C6—H6	0.9300	C14—C15	1.363 (10)
C7—H7A	0.9600	C14—H14	0.9300
C7—H7B	0.9600	C15—H15	0.9300
C7—H7C	0.9600	C16—H16A	0.9600
C8—H8A	0.9600	C16—H16B	0.9600
C8—H8B	0.9600	C16—H16C	0.9600
C8—H8C	0.9600	N1—H1	0.860 (5)
			
C2—C1—C6	120.5 (5)	O1—C9—N1	123.8 (6)
C2—C1—N1	119.8 (5)	O1—C9—C10	121.2 (5)
C6—C1—N1	119.7 (5)	N1—C9—C10	115.0 (5)
C1—C2—C3	119.6 (5)	C11—C10—C15	121.0 (5)
C1—C2—C7	120.5 (6)	C11—C10—C9	120.8 (5)
C3—C2—C7	119.9 (6)	C15—C10—C9	118.0 (6)
C2—C3—C4	118.1 (6)	C12—C11—C10	117.2 (6)
C2—C3—C8	120.5 (7)	C12—C11—C16	119.1 (6)
C4—C3—C8	121.4 (7)	C10—C11—C16	123.7 (6)
C5—C4—C3	121.0 (6)	C11—C12—C13	122.6 (7)
C5—C4—H4	119.5	C11—C12—H12	118.7
C3—C4—H4	119.5	C13—C12—H12	118.7
C4—C5—C6	121.4 (6)	C14—C13—C12	120.0 (7)
C4—C5—H5	119.3	C14—C13—H13	120.0
C6—C5—H5	119.3	C12—C13—H13	120.0
C5—C6—C1	119.3 (6)	C15—C14—C13	118.7 (7)
C5—C6—H6	120.3	C15—C14—H14	120.6
C1—C6—H6	120.3	C13—C14—H14	120.6
C2—C7—H7A	109.5	C14—C15—C10	120.4 (6)
C2—C7—H7B	109.5	C14—C15—H15	119.8
H7A—C7—H7B	109.5	C10—C15—H15	119.8
C2—C7—H7C	109.5	C11—C16—H16A	109.5
H7A—C7—H7C	109.5	C11—C16—H16B	109.5
H7B—C7—H7C	109.5	H16A—C16—H16B	109.5
C3—C8—H8A	109.5	C11—C16—H16C	109.5
C3—C8—H8B	109.5	H16A—C16—H16C	109.5
H8A—C8—H8B	109.5	H16B—C16—H16C	109.5
C3—C8—H8C	109.5	C9—N1—C1	125.3 (5)
H8A—C8—H8C	109.5	C9—N1—H1	121 (5)
H8B—C8—H8C	109.5	C1—N1—H1	108 (5)
			
C6—C1—C2—C3	1.5 (8)	N1—C9—C10—C15	−49.1 (7)
N1—C1—C2—C3	−178.5 (5)	C15—C10—C11—C12	0.2 (9)
C6—C1—C2—C7	−178.0 (6)	C9—C10—C11—C12	177.0 (6)
N1—C1—C2—C7	2.0 (8)	C15—C10—C11—C16	−179.2 (6)
C1—C2—C3—C4	−2.6 (9)	C9—C10—C11—C16	−2.4 (9)
C7—C2—C3—C4	176.9 (6)	C10—C11—C12—C13	0.5 (11)
C1—C2—C3—C8	175.6 (6)	C16—C11—C12—C13	179.9 (8)
C7—C2—C3—C8	−4.9 (9)	C11—C12—C13—C14	−1.0 (13)
C2—C3—C4—C5	2.4 (10)	C12—C13—C14—C15	0.8 (12)
C8—C3—C4—C5	−175.9 (7)	C13—C14—C15—C10	−0.2 (11)
C3—C4—C5—C6	−0.9 (12)	C11—C10—C15—C14	−0.3 (10)
C4—C5—C6—C1	−0.3 (11)	C9—C10—C15—C14	−177.2 (6)
C2—C1—C6—C5	0.0 (9)	O1—C9—N1—C1	−1.4 (10)
N1—C1—C6—C5	180.0 (6)	C10—C9—N1—C1	179.7 (6)
O1—C9—C10—C11	−45.0 (9)	C2—C1—N1—C9	−134.7 (6)
N1—C9—C10—C11	133.9 (6)	C6—C1—N1—C9	45.3 (8)
O1—C9—C10—C15	132.0 (7)		

### Hydrogen-bond geometry (Å, °)

**Table:**

*D*—H···*A*	*D*—H	H···*A*	*D*···*A*	*D*—H···*A*
N1—H1···O1^i^	0.86 (1)	2.23 (5)	2.903 (6)	136 (6)

Symmetry codes: (i) *x*, *y*+1, *z*.
